# Impact of fracture fixation surgery on cognitive function and the gut microbiota in mice with a history of stroke

**DOI:** 10.1515/biol-2022-1061

**Published:** 2025-02-25

**Authors:** Yu Lu, Zixuan Li, Rukun Xu, Yajie Xu, Wenwen Zhang, Yong Zhang, Zhaojing Fang, Cailong Pan, Xiaoliang Wang

**Affiliations:** Department of Anesthesiology, Perioperative and Pain Medicine, Nanjing First Hospital, Nanjing Medical University, Changle Road 68, Nanjing, 210029, China; School of Basic Medical Sciences, Nanjing Medical University, Longmian Avenue 101, Nanjing, 211166, China

**Keywords:** cognitive dysfunction, fracture, stroke, gut microbiota

## Abstract

Perioperative cognitive dysfunction is a common complication in stroke patients undergoing secondary surgeries. This study investigated the effects of tibial fracture internal fixation (TFIF) surgery on cognitive function and the gut microbiota in mice with a history of stroke. Using the middle cerebral artery occlusion method to induce stroke, we assessed cognitive function via the fear conditioning test and analyzed the gut microbiota through 16S rRNA sequencing. Compared with those in the normal and stroke groups, the cognitive function of the mice in the stroke group that underwent TFIF surgery was significantly impaired. Gut microbiota analysis revealed significant changes in beta diversity, but not in alpha diversity, in these mice. Additionally, TFIF surgery increased microglial activation and IL-1β and lipopolysaccharide (LPS) levels in the brain while reducing α-defensin levels and increasing IL-1β and LPS levels in the colon. These results suggest that TFIF surgery exacerbates cognitive impairment in stroke mice, possibly through alterations in the gut microbiota that impair intestinal defense and promote inflammation. This study highlights the critical role of the gut microbiome in cognitive function and perioperative outcomes, offering insights into potential therapeutic strategies for perioperative cognitive dysfunction in stroke patients.

## Graphical abstract

**Figure:**
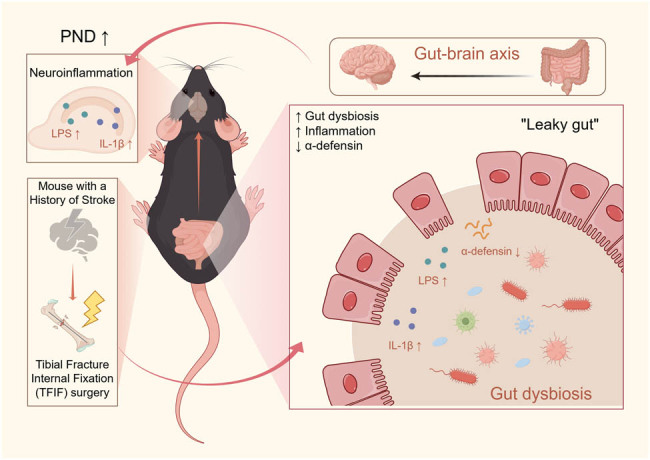

## 1 Introduction

Perioperative neurocognitive disorder (PND) encompasses a range of cognitive function changes that occur before and/or after surgery, significantly impacting patients’ quality of life, hospitalization duration, and mortality rates [1]. Surgeries, particularly those involving anesthesia and trauma, have been shown to influence cognitive function through mechanisms such as neuroinflammation, oxidative stress, and disruption of the blood‒brain barrier [2–4]. These effects are especially pronounced in vulnerable populations, including stroke survivors. Stroke survivors face an increased risk of bone fractures [5], and the incidence of stroke in young individuals is increasing [6,7]. These trends suggest a growing need for bone fixation surgical treatments in stroke patients. However, the impact of bone fixation surgery on cognitive impairment in stroke patients remains largely unexplored.

The gut microbiome is a complex microbial system that can affect brain function and behavior through neural, endocrine, and immune pathways, forming a bidirectional information exchange network with the brain [8–10]. Dysbiosis of the gut microbiome has been implicated in the progression of cognitive dysfunction, including PND [11]. Changes in the gut microbiome can regulate neuroinflammatory processes, which in turn affect cognitive outcomes [12–14]. Previous studies have demonstrated a connection between dysbiosis of the gut microbiome and the progression of PND. Additionally, surgery under general anesthesia has been associated with “leaky gut,” characterized by increased intestinal permeability and gut microbiome dysbiosis [15–17]. However, the impact of fracture fixation surgery after stroke on the composition of the gut microbiome and its relationship with the occurrence of PND remains unclear, necessitating further investigation.

Therefore, the aim of this study was to determine the changes in cognitive function and their correlation with the gut microbiome before and after fracture fixation surgery under normal and stroke conditions. To achieve this objective, in the present study, mice with or without stroke underwent fracture fixation surgery, and their cognitive function and gut microbiome were detected. This study may have the potential to advance our understanding of the complex interplay between the gut microbiome and cognitive function in stroke patients undergoing surgery.

## 2 Materials and methods

### 2.1 Reagents

Pentobarbital sodium (No. WS20140220), chromatography-grade methanol (No. 138507), chromatography-grade acetonitrile (No. 173534), and chromatography-grade formic acid (No. 172433) were purchased from Thermo Fisher Scientific (China) Co., Ltd. The QIAamp DNAStool Mini Kit was obtained from QIAGEN, and the AxyPrepDNA Gel Recovery Kit was purchased from AXYGEN Corporation.

### 2.2 Experimental design and treatment of mice

Healthy 8-week-old male C57BL/6 mice were obtained from Shanghai SLAC Laboratory Animal Co., Ltd. All experimental protocols and procedures were approved and licensed by the Nanjing Medical University Animal Care and Use Committee in accordance with the National Institutes of Health Guide for the Care and Use of Laboratory Animals (approval number: 2207037). The mice were housed in the SPF-grade animal facility at the Animal Experimental Center of Nanjing Medical University with a 12 h light‒dark cycle, a temperature of 25 ± 1°C, and relative humidity ranging from 40 to 60%.

The C57BL/6 mice were randomly divided into two groups: the control group (NC) and the middle cerebral artery occlusion (MCAO) group. After 1 week of acclimation, the mice in the MCAO group underwent the modified Z-longa suture method to induce MCAO, whereas those in the control group (NC) received no surgical treatment but were subjected to the same conditions as the MCAO mice. Fear conditioning tests (FCTs) were conducted on the 14th and 23rd days after stroke induction. On the 23rd day after stroke, both the control group and the MCAO group experienced tibial bone fracture and intramedullary fixation surgery. FCTs were repeated on the third and seventh days after surgery for both groups.


**Ethical approval:** The research related to animal use has been complied with all the relevant national regulations and institutional policies for the care and use of animals, and has been approved by the Nanjing Medical University Animal Care and Use Committee in accordance with the National Institutes of Health guide for the care and use of Laboratory animals (approval number: 2207037).

### 2.3 Replication of the mouse stroke model

After 7 days of adaptive feeding, a stroke model was induced in C57BL/6 mice. In the model group, the mice were subjected to suturing to induce MCAO. A suture was inserted into the left external carotid artery and advanced through the internal carotid artery. The insertion was stopped and fixed upon encountering resistance. After 45 min, the suture was removed to allow for reperfusion. The criteria for successful model induction were as follows: the mice exhibited conscious left limb paralysis, unsteady posture, and circling toward one side when their tail was lifted.

### 2.4 Neurological scoring

Neurological function impairment following ischemic injury was evaluated in a blinded manner using the Longa scoring system at 72 h post-injury. The scoring system was as follows: 0 = normal, 1 = failure to fully extend the left forelimb, 2 = circling to the left during walking, 3 = leaning to the left during walking, and 4 = inability to ambulate spontaneously and loss of consciousness. Additionally, if the mouse failed to perform tasks such as flexion of the forelimbs, flexion of the hindlimbs, and moving the head >10° off the vertical axis within 30 s when lifted by the tail, an additional score of 1 was assigned.

### 2.5 Tibial fracture internal fixation (TFIF) surgery

The mice were subjected to intramedullary fixation surgery for tibial fracture under isoflurane anesthesia, following the procedure described in a previous study [18]. Briefly, the mice were anesthetized with isoflurane (2.0% isoflurane, 0.30 FiO_2_) and maintained throughout the procedure. A skin incision was made below the knee to expose the tibia. A 0.3 mm needle was inserted into the medullary cavity of the tibia for intramedullary fixation. A fracture was subsequently created at the midpoint of the tibia using scissors. Finally, the wound was thoroughly cleaned and sutured. The animals were then allowed to recover from anesthesia. Throughout the procedure, a warming pad was used to maintain the body temperature of the mice at approximately 37°C. Postanesthesia, a single dose of buprenorphine (0.4 mg/kg) was administered subcutaneously for analgesic treatment prior to skin closure.

### 2.6 FCTs

FCTs were conducted on the 14th and 23rd days after stroke induction, as well as on the 3rd and 7th days after TFIF. Each mouse was acclimated to the conditioned fear chamber for 120 s, followed by exposure to an auditory stimulus (3.6 kHz, 70 dB) and an electric foot shock during the last 2 s of the auditory stimulus (2 s, 0.8 mA). This combined auditory‒foot shock training was repeated three times with a 60 s interval between each training session. After the three training sessions, the mice were removed from Chamber A after 1 min. Twenty-four hours later, contextual FCT was conducted. Each mouse was placed back into the conditioned fear chamber, where it was trained, and observed for 6 min without any auditory or electric shock stimulation. Freezing behavior was scored during this period. Two hours after the contextual FCT, the auditory-conditioned fear test was performed in a new experimental chamber. Each mouse was placed in the chamber and allowed to explore for 3 min. The auditory stimulus was subsequently presented for 3 min, and freezing behavior was recorded. Freezing behavior was defined as the absence of all visible movements except for respiration and was assessed using an automated video tracking system (ACT-100A; Coulbourn, USA).

### 2.7 2,3,5-Triphenyltetrazolium chloride (TTC) staining

To determine the size of the infarction, the whole brains of the mice were stained with TTC to obtain 2 mm-thick brain slices. The infarct size was quantified using i-Solution software (Image and Microscope Technology, Vancouver, Canada).

### 2.8 Ionized calcium binding adaptor molecule 1 (IBA1) immunohistochemical staining of mouse brain tissue

As previously described [19], 5 μm-thick paraffin-embedded coronal sections of the hippocampus were mounted on Superfrost microscope slides. Antigen retrieval was performed by incubating the sections in Tris/EDTA buffer (10 mM Tris base, 1 mM EDTA, 0.05% Tween 20, pH 9.0) at 95–100°C for 20 min. After being washed in Tris-buffered saline (TBS) containing 0.025% Triton X-100, the sections were blocked in 10% donkey serum with 1% bovine serum albumin in TBS for 2 h, followed by overnight incubation at 4°C with a primary rabbit polyclonal anti-ionized calcium-binding adapter molecule 1 (Iba-1) antibody (1:500, 019–19741; Wako Chemicals, USA). The sections were subsequently incubated with the appropriate secondary antibody and nuclear stain. The assembled slice images were captured using a microscope (Olympus BX43). Three independent microscopic fields were randomly obtained in the dentate gyrus region of each slice using a counting frame size of 0.4 mm, and three slices per mouse were imaged. Quantification was performed using ImageJ (National Institutes of Health, Bethesda, USA). The level of positive immunoreactivity was evaluated by the average optical density of positively stained areas. The average optical density of these identified positive regions was then calculated, with multiple measurements taken across different fields of the sample.

### 2.9 ELISA detection

ELISA was performed to measure the levels of the inflammatory cytokines IL-1β and lipopolysaccharide (LPS) in the hippocampal tissue and colonic mucosa of each group of mice. The specific procedures were conducted strictly according to the instructions provided with the ELISA kit.

### 2.10 Detection and analysis of the mouse fecal microbiota

Fresh fecal pellets were collected 48 h after MCAO and tibial bone fracture with intramedullary fixation surgery. The samples were immediately flash-frozen in liquid nitrogen and stored at −80°C for future analysis. High-throughput sequencing and analysis of the mouse fecal samples were subsequently performed. The microbial community total DNA was extracted using the CTAB method from various microbial community samples from different sources. The quality of the extracted DNA was assessed by agarose gel electrophoresis, and DNA quantification was performed using a UV spectrophotometer. High-throughput sequencing analysis was conducted on the V4–V5 region of the 16S rDNA gene. Using purified DNA as a template, PCR amplification was performed on the target fragment of the 16S rDNA V4–V5 region using the universal primers 515F (5′-GTGCCAGCMGCCGCGG-3′) and 926R (5′-CCGTCAATTCMTTTGAGTTT-3′). The PCR products were detected by 2% agarose gel electrophoresis, and the products were recovered using AMPure XT beads. The purified PCR products were evaluated using an Agilent 2100 Bioanalyzer (Agilent, USA) and a library quantification kit from Illumina (KapaBiosciences, Woburn, MA, USA). Qualified libraries were gradient diluted and mixed in the appropriate proportions based on the desired sequencing volume and then denatured with NaOH to generate single-stranded templates for sequencing. Sequencing was performed on a NovaSeq 6000 sequencer using a 2 × 250 bp paired-end sequencing protocol and a NovaSeq 6000 SP Reagent Kit (500 cycles). After all the raw data were screened, optimized sequences were obtained and subjected to operational taxonomic unit clustering. Alpha diversity analysis and beta diversity analysis were performed on the basis of the obtained ASV (feature) sequences and ASV (feature) abundance tables. Alpha diversity analysis was used to evaluate the diversity within the habitat using six indices: Chao1, observed_species, Shannon, Simpson, goods_coverage, and pielou_e. Beta diversity analysis was used to assess the diversity between habitats (samples/groups) using four distances (weighted_unifrac, unweighted_unifrac, jaccard, bray_curtis) and six analyses. PCoA and NMDA analyses were conducted using the R language (version 3.2.3). The microbiota at different taxonomic levels were defined, and their relative abundances were calculated. Different types of visualization graphics were used to display and describe the data. Welch’s *t* test in the STAMP software was used to filter out parts with *P* ≥ 0.05. The functional differences between different groups were analyzed using the PICRUSt2 software (https://github.com/picrust/picrust2).

### 2.11 Immunohistochemical staining for α-defensin

Mouse colon tissues were harvested on the seventh day following the intramedullary fixation of tibial fractures. These tissues were fixed in paraformaldehyde after being rinsed with saline, followed by dehydration using sucrose postfixation. The tissues were subsequently embedded in paraffin and sectioned. The sections were dewaxed and rehydrated through a series of alcohol gradients. To block nonspecific binding, the sections were incubated in 10% donkey serum plus 1% bovine serum albumin in TBS for 2 h. The samples were then incubated overnight at 4°C with a primary antibody specific for α-defensin. After thorough washing, the sections were incubated with a biotinylated secondary antibody, followed by the addition of an avidin–biotin–peroxidase complex. The immunoreactivity was visualized using a chromogenic substrate. Counterstaining was performed with hematoxylin to highlight the tissue morphology. The sections were dehydrated, cleared, and mounted with permanent mounting medium. The level of positive immunoreactivity was evaluated by assessing the intensity and distribution of the brown-stained areas under a light microscope (Olympus BX43).

### 2.12 Statistical analyses

In addition to the partial intestinal microbiota sequencing results, the other quantitative data were subjected to statistical analysis using GraphPad Prism 9.0 software. The results are presented as the means ± standard deviations. The differences between two groups were analyzed via *t* tests, with *P* < 0.05 considered statistically significant. Multiple group comparisons were performed using one-way analysis of variance, with *P* < 0.05 indicating statistical significance.

## 3 Results

### 3.1 TFIF increased brain inflammation and promoted cognitive dysfunction in stroke model mice

**Figure 1:**
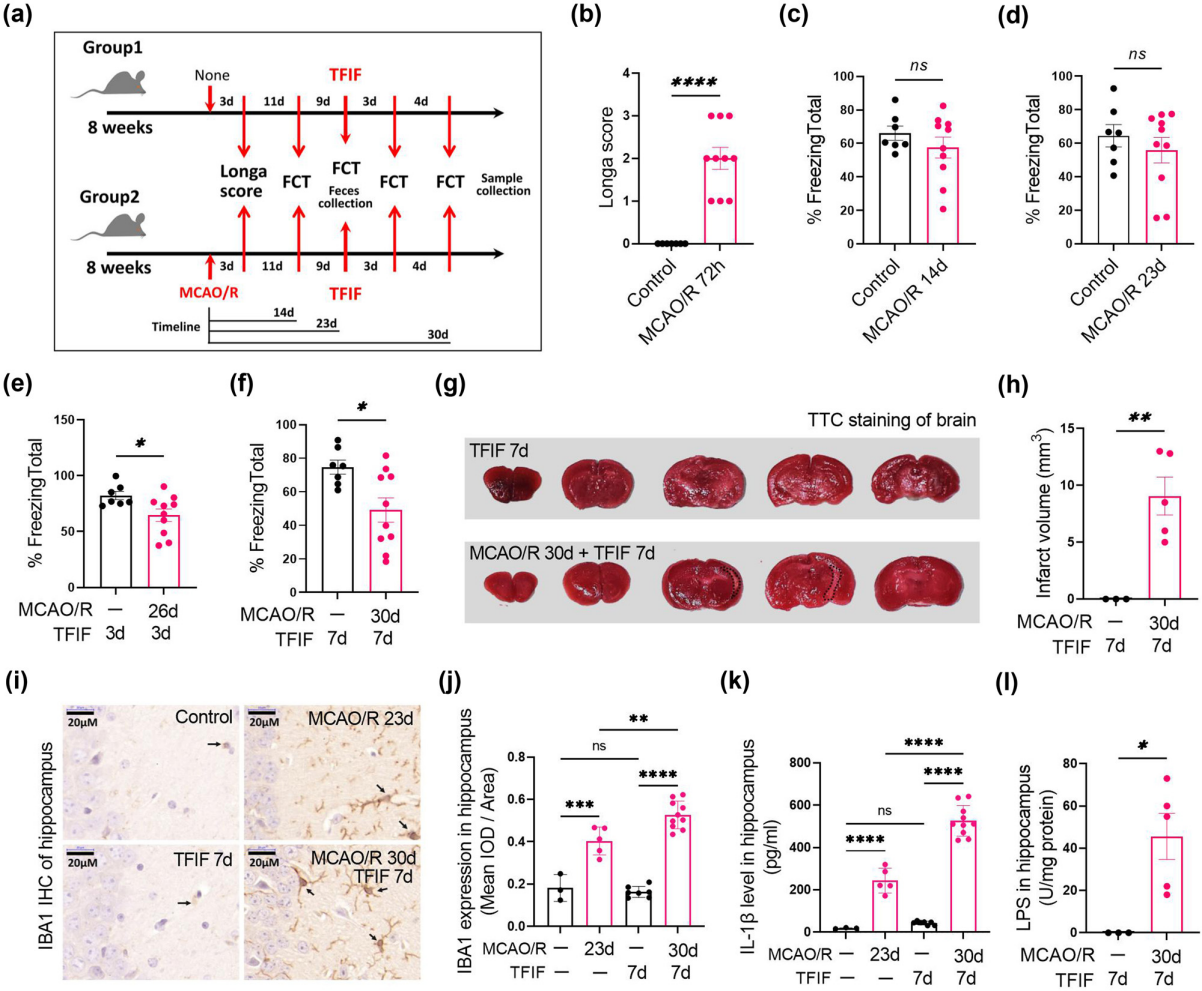
TFIF increases brain tissue inflammation and impairs cognitive function in mice with stroke. (a) Schematic representation of mouse grouping and different interventions, including stroke timing, TFIF procedure, and timing of laboratory assessments. (b) Neurological assessment scores of mice 72 h after stroke. (c) FCT to evaluate memory function in mice 14 days after stroke. (d) FCT to evaluate memory function in mice 23 days after stroke. (e) FCT to evaluate memory function in mice 3 days after TFIF performed 23 days after stroke (26 days after stroke). (f) FCT to evaluate memory function in mice 7 days after TFIF performed 23 days after stroke (30 days after stroke). (g) and (h) TTC staining to assess brain infarct volume in mice undergoing TFIF 7 days after stroke (30 days after stroke). (i) and (j) Immunohistochemical staining of IBA1 in mouse brain tissue and analysis of average optical density. (k) ELISA measurement of IL-1β levels in mouse hippocampal tissue. (l) ELISA measurement of LPS levels in mouse hippocampal tissue.

First, we investigated the effects of TFIF surgery on brain tissue inflammation and cognitive impairment in mice with stroke. The experimental timeline and procedures are illustrated in Figure 1a. The mice exhibited stroke-related sensorimotor deficits 72 h after stroke induction (Figure 1b). Cognitive function was assessed using a FCT at 14 and 23 days poststroke, revealing recovery of cognitive function in stroke mice after 2 weeks (Figure 1c and d). TFIF surgery was performed on stroke mice 23 days poststroke, and the results were compared with those of nonstroke mice that underwent the same surgery. The stroke mice presented a significant reduction in freezing time on the third day after TFIF surgery (Figure 1e), which was lower than that of the surgery-only mice without a history of stroke on the seventh day (Figure 1f). TTC staining was used to evaluate the brain infarct volume in stroke and nonstroke mice at 7 days post-TFIF surgery, which was performed 30 days poststroke. The results revealed the presence of an infarct area in the brain (Figure 1g and h). In normal mice that underwent TFIF surgery for 7 days, there was no significant activation of hippocampal microglia. Stroke is a significant factor that causes microglial activation in mouse brain tissue, as evidenced by a significant increase in the number of microglia in the hippocampal region of both stroke and nonstroke mice. Notably, TFIF surgery further promoted microglial activation in young mice with stroke (Figure 1i and j). The changes in the levels of IL-1β in the hippocampal tissue of the mice were similar to the changes in the IBA1 expression levels among the different groups (Figure 1k). Only the hippocampal tissue of stroke model mice that underwent TFIF surgery presented detectable levels of LPS (Figure 1l).

### 3.2 TFIF alters the β-diversity of gut microbiota in mice with stroke

**Figure 2:**
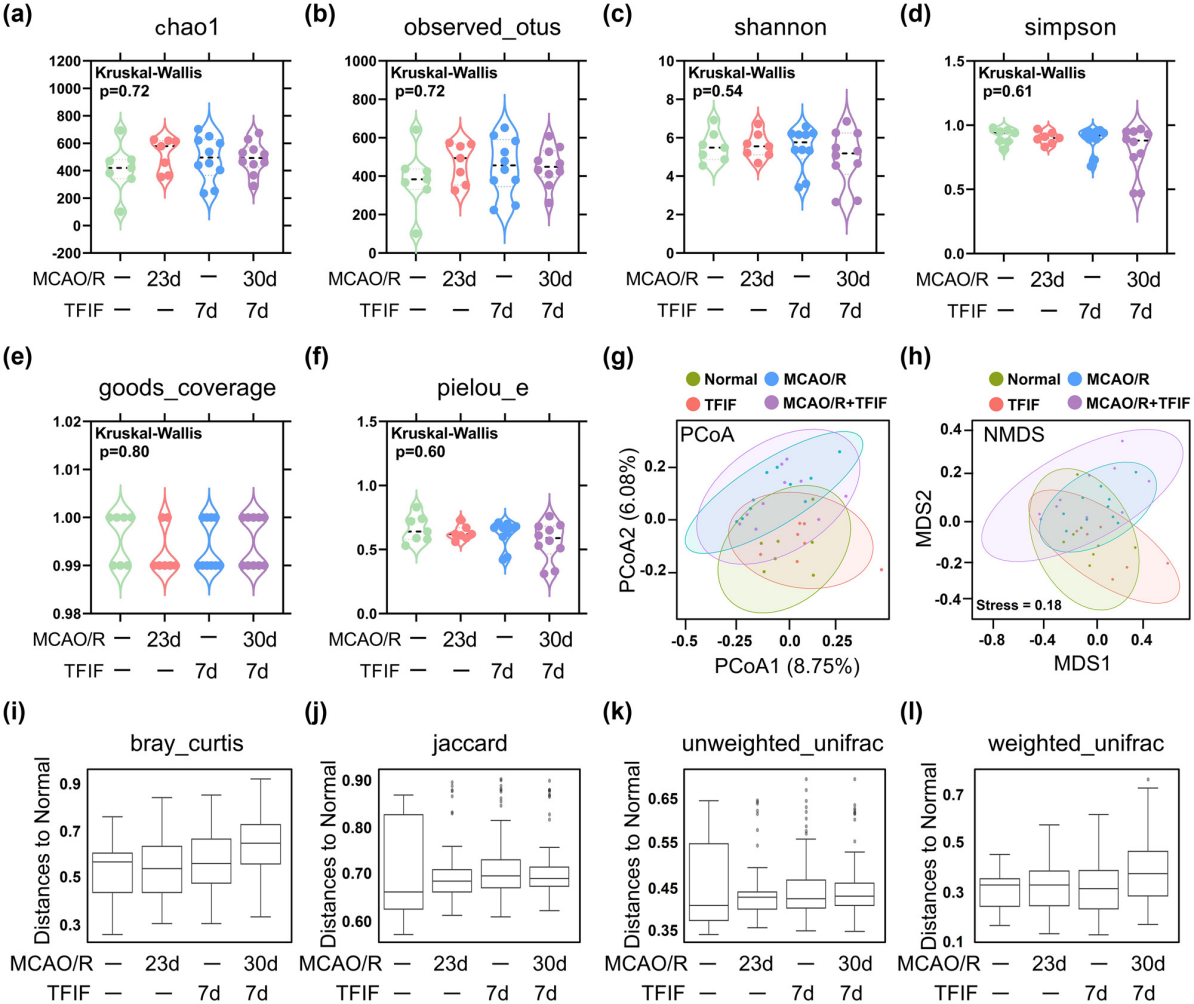
TFIF alters β-diversity of gut microbiota in mice with stroke. (a)–(f) Assessment of α-diversity of gut microbiota composition in mouse feces using chao1, observed_species, shannon, simpson, goods_coverage, and pielou_e indices. (g) PCoA of gut bacteria in mice. (h) NMDS of gut bacteria in mice. (i)–(l) Analysis of similarities to evaluate the dissimilarity among groups. The distances between the gut microbiota composition of different groups and the normal group were calculated using four different indices: bray_curtis, jaccard, unweighted_unifrac, and weighted_unifrac. In the normal group and MCAO/R 23d group, *n* = 7. In the TFIF groups, *n* = 10.

We next examined whether the gut microbiota of mice with a history of stroke changed after TFIF surgery. The α-diversity refers to the diversity of species and bacteria within a community or habitat, whereas the β-diversity represents the differentiation among habitats. The Chao1 and observed species indices were used to characterize richness, the Shannon and Simpson indices were used to represent diversity, Pielou’s evenness index was used to represent evenness, and Good’s coverage index was used to represent coverage. There were no significant changes in α-diversity observed in the mice before or after TFIF (Figure 2a–f). Furthermore, principal coordinate analysis (PCoA) and non-metric multidimensional scaling (NMDS) analysis revealed that the impact of TFIF on the β-diversity of the gut microbiota was greater than the impact of stroke on the mouse microbiota. In both analyses, the two groups of mice subjected to TFIF were separated from the two groups of mice not subjected to TFIF, but the influence of stroke had little effect on the separation of the microbiota (Figure 2g and h). The distances between the gut microbiota compositions of the mice and the normal group were calculated via Bray‒Curtis, Jaccard, unweighted UniFrac, and weighted UniFrac metrics. Among the four different statistical algorithms, with the exception of the Bray‒Curtis metric, the other calculations revealed that the mice receiving TFIF after stroke had the greatest distance from the gut microbiota of normal young mice (Figure 2i–l). Jaccard and unweighted UniFrac analysis also revealed that stroke or TFIF alone led to the separation of the microbiota from the normal group (Figure 2i–l).

### 3.3 TFIF alters the composition of the gut microbiota at different taxonomic levels in mice with stroke

**Figure 3:**
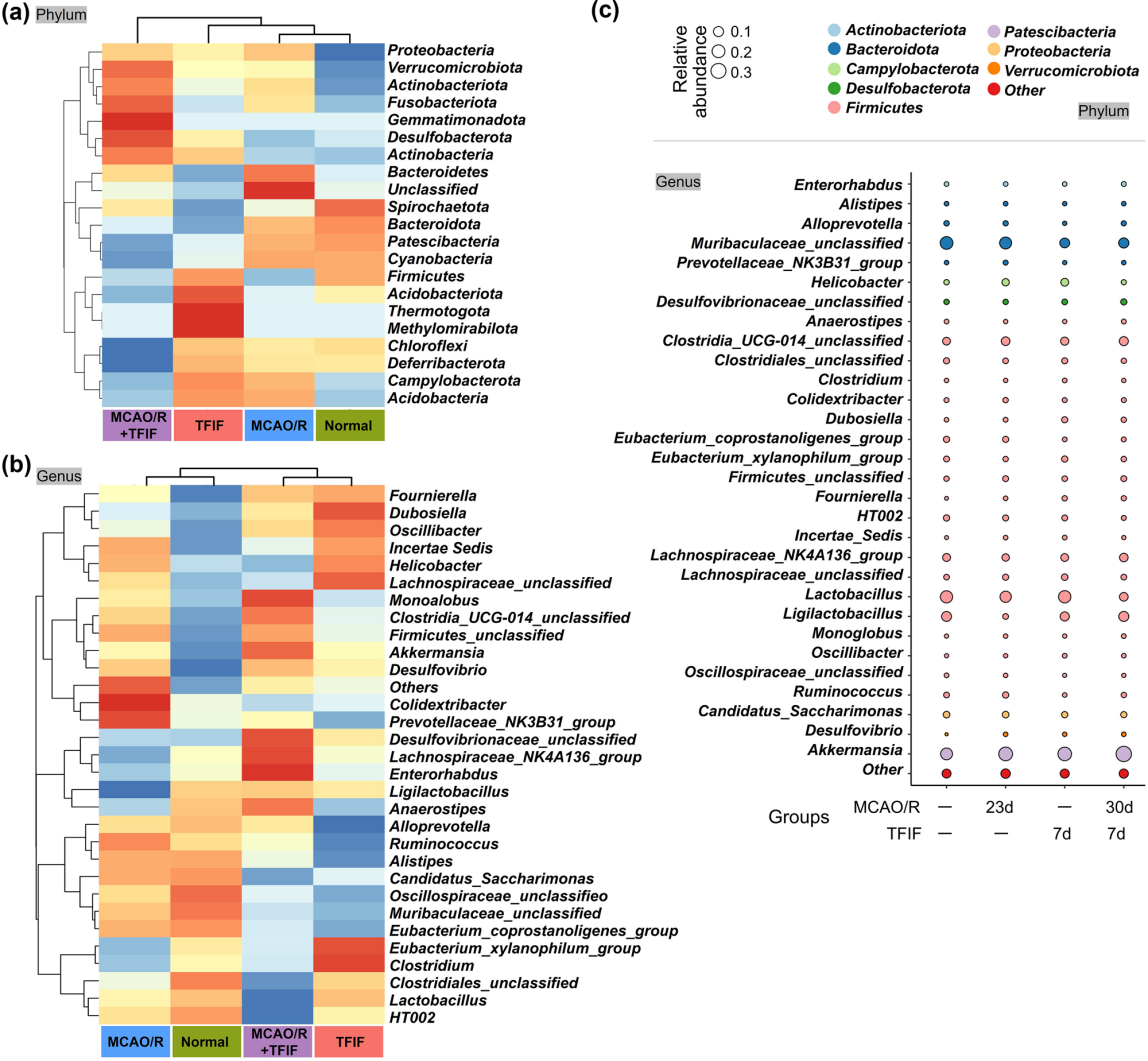
TFIF alters the composition of gut microbiota at different taxonomic levels in mice with stroke. (a) Heatmaps displaying the composition of gut bacteria at the phylum level in the four groups of mice. (b) Heatmaps displaying the composition of gut bacteria at the genus level in the four groups of mice. (c) Bubble plot illustrating the phyla to which the different genera-level gut bacteria in the feces of mice belong, along with their relative expression.

The relative abundances of phyla and genera of the gut microbiota in the feces of the control, TFIF, stroke, and stroke with TFIF groups were visualized using a heatmap and bubble plot. The relative expression abundance of 21 phyla-level gut microbiota in different groups is displayed, and both taxa and groups were subjected to cluster analysis. At the phylum level, the MCAO/R group was the closest to the normal group, followed by the TFIF group, and the MCAO/R + TFIF group presented the most distinct pattern (Figure 3a). The heatmap at the genus level displayed the relative abundance of the top 30 species (Figure 3b). The bubble plot represents the phyla to which the top 30 species at the genus level belong, further highlighting their relative differences among the groups. These predominantly enriched phyla included Actinobacteriota, Bacteroidota, Campylobacterota, Desulfobacterota, Firmicutes, Patescibacteria, Proteobacteria, and Verrucomicrobiota (Figure 3c).

### 3.4 Identification of representative species with altered gut microbiota composition in mice with stroke after TFIF

**Figure 4:**
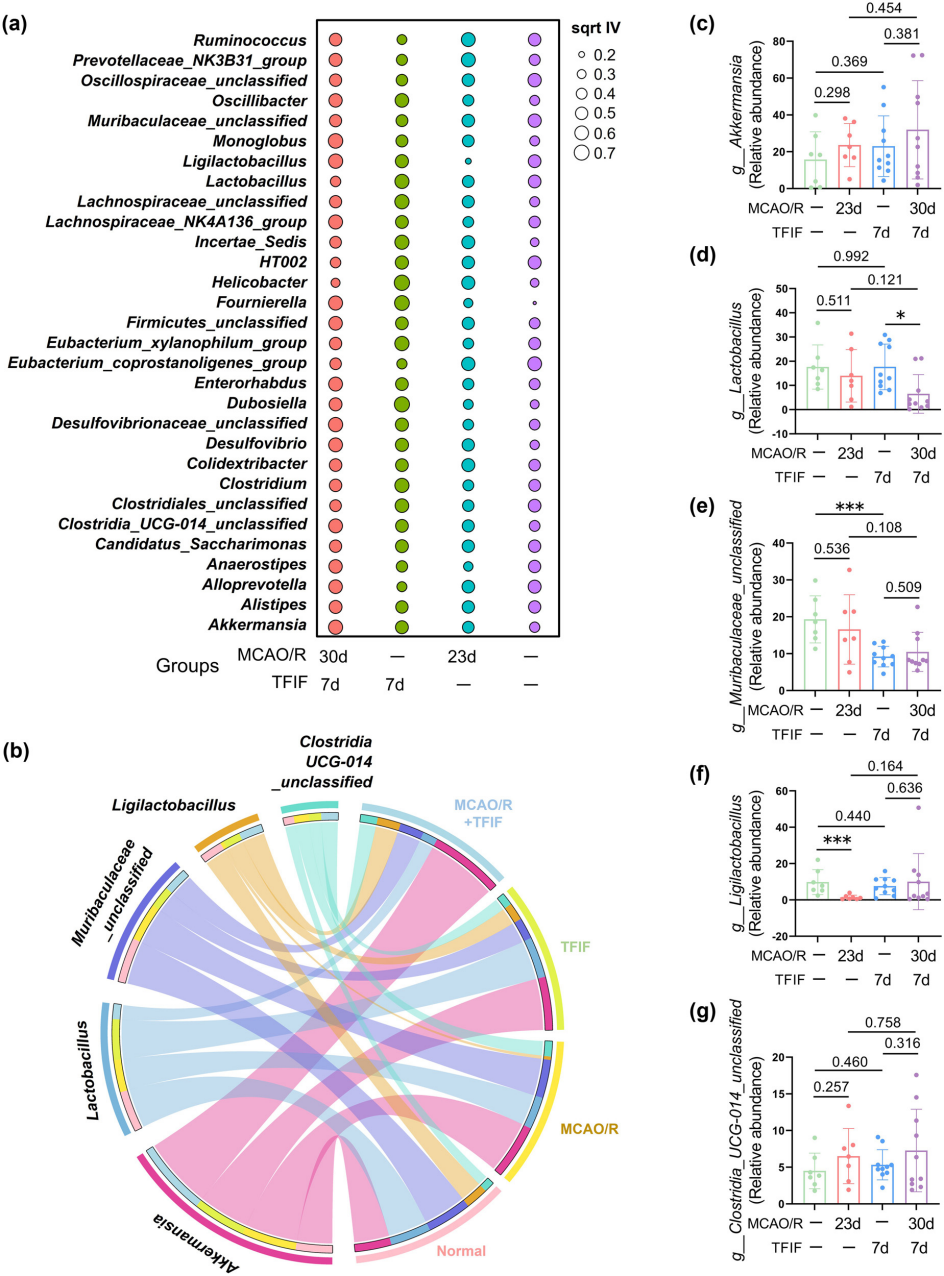
Selection of representative species with altered gut microbiota composition in young mice after TFIF and stroke. (a) Bubble plot generated from the phylum-level indicator analysis indicates the top 30 abundant species, where the square root of the indicator value (sqrt IV) is represented. (b) Circular plot showing the top five dominant species at the genus level in the gut microbiota of the four groups of mice, arranged in descending order of abundance on the left, with grouping information on the right. (c)–(g) Differential analysis of species abundance profiles extracted from the top five dominant species (genus level) in the fecal samples of the four groups of mice. The species include *Akkermansia*, *Lactobacillus*, Muribaculaceae_unclassified, Ligilactobacillus, and Clostridia_UCG-014_unclassified. In the normal group and MCAO/R 23d group, *n* = 7. In the TFIF groups, *n* = 10.

Representative species analysis is a classical analysis method for amplicon data that identifies significant biomarkers. At the genus level, we performed an analysis using the 30 most abundant species to identify potential biomarkers. Among the genera, the control group presented relatively greater abundances of Eubacterium_coprostanoligenes_group, Lactobacillus, Muribaculaceae_unclassified, Ligilactobacillus, Clostridiales_unclassified, HT002, Ruminococcus, Alloprevotella, and Oscillospiraceae_unclassified. The TFIF group presented greater abundances of Prevotellaceae_NK3B31_group, Ruminococcus, Muribaculaceae_unclassified, Helicobacter, Eubacterium_coprostanoligenes_group, Colidextribacter, Alloprevotella, and Incertae_Sedis. The stroke group presented increased abundances of Helicobacter, Dubosiella, Fournierella, Lachnospiraceae_unclassified, Eubacterium_xylanophilum_group, Lactobacillus, Clostridium, and Incertae_Sedis. The poststroke TFIF group presented increased abundances of Monoglobus, Ligilactobacillus, Desulfovibrionaceae_unclassified, Akkermansia, Lachnospiraceae_NK4A136_group, Fournierella, Enterorhabdus, and Desulfovibrio (Figure 4a).

Dominant species play crucial roles in the functioning of the gut microbiota. To further elucidate which species are involved in cognitive impairment following stroke and fracture, we selected the top five dominant species within each group on the basis of their abundance and presented them using a circular plot. The identified dominant species were Akkermansia, Lactobacillus, Muribaculaceae_unclassified, Ligilactobacillus, and Clostridia_UCG-014_unclassified (Figure 4b). Among them, Akkermansia and Clostridia_UCG-014_unclassified did not significantly differ among the groups. Lactobacillus exhibited a significant decrease in abundance in the stroke mice treated with TFIF, whereas TFIF significantly reduced the abundance of Muribaculaceae_unclassified in the nonstroke mice. Stroke significantly decreased the abundance of Ligilactobacillus in mice (Figure 4c).

### 3.5 TFIF impaired intestinal defenses and promoted intestinal inflammation in stroke mice

**Figure 5:**
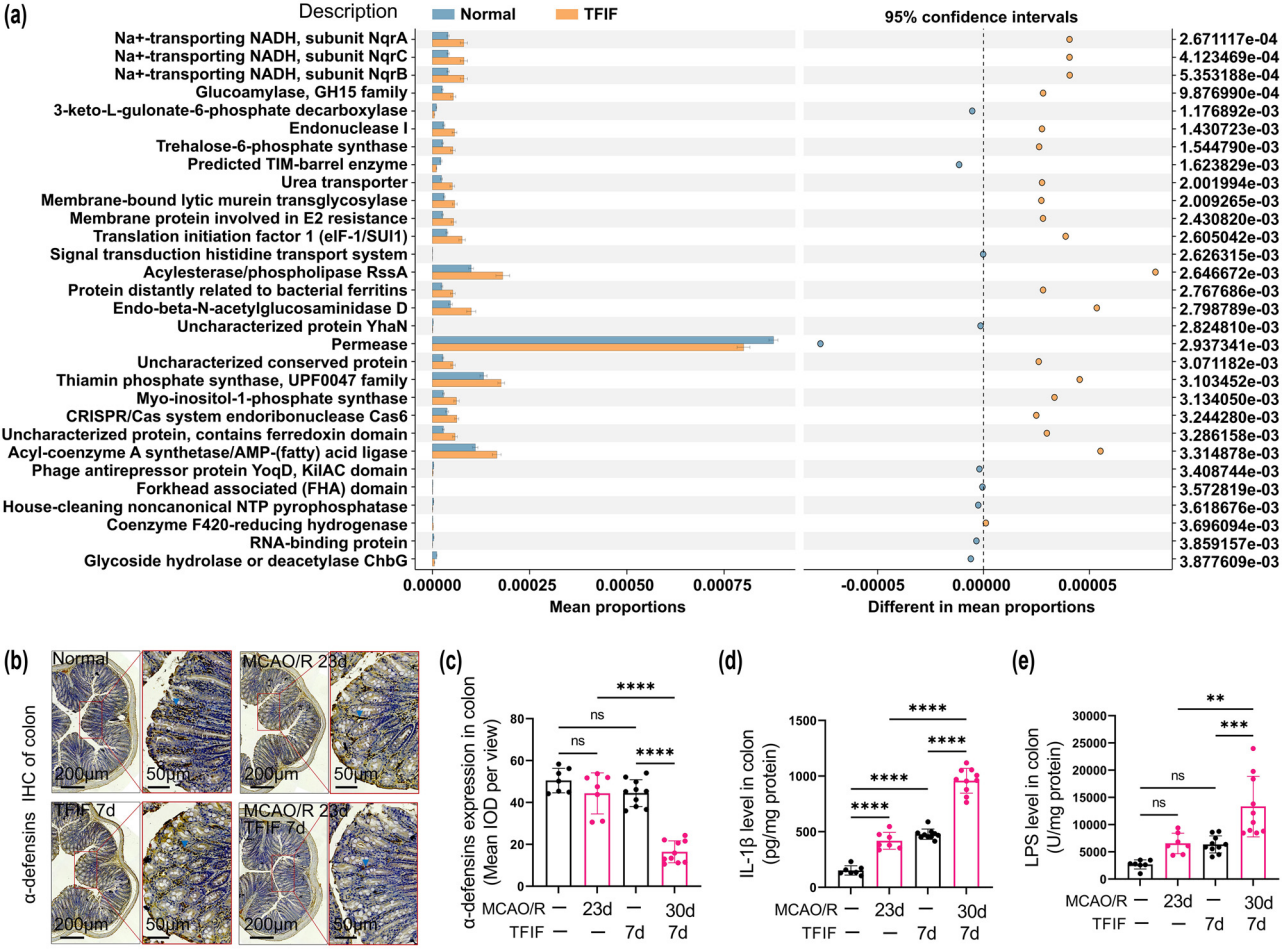
Alteration of intestinal defense function and promotion of intestinal inflammation in mice with TFIF and stroke. (a) Functional prediction of differentially abundant microbiota between the normal group and the TFIF surgery group. STAMP differential analysis and COG database analysis were performed to investigate the functional relevance of the microbiota. The left panel shows the description of the associated functions, the upper panel indicates the 95% confidence interval, and the right panel displays the *P*-values. (b) and (c) Immunohistochemistry staining and analysis of average pixel intensity for α-defensin in mouse colonic tissues. (d) ELISA measurement of IL-1β levels in the colonic mucosal homogenate of mice. (e) ELISA measurement of LPS levels in the colonic mucosal homogenate of mice. In the normal group and MCAO/R 23d group, *n* = 7. In the TFIF groups, *n* = 10.

The functional differences between different groups were analyzed using the PICRUSt2 software (https://github.com/picrust/picrust2), and a predicted functional analysis of the gut microbiota was conducted. The results revealed significant alterations in the microbiota between the normal group and the TFIF group. The microbiota was found to be involved in various biological functions, with the highest abundance of permease among the top 30 significantly altered functions (Figure 5a). After the levels of colonic α-defensins in the mice were evaluated, neither stroke alone nor TFIF alone caused a decrease in the colonic α-defensin level. However, in the mice that experienced stroke and received TFIF surgery for 7 days, a significant reduction in the colonic α-defensin level was observed (Figure 5b and c). The colonic IL-1β levels in the mice showed that stroke alone or TFIF alone was sufficient to increase the levels of IL-1β in the gut, while the mice that underwent stroke combined with TFIF surgery presented the highest levels of IL-1β (Figure 5d). Compared with mice subjected to stroke alone or TFIF alone, mice that received TFIF after stroke presented significantly greater LPS levels in the colonic villi (Figure 5e).

## 4 Discussion

The risk of fracture is significantly increased after a stroke, and fracture internal fixation surgery is the most common procedure performed after a fracture [20,21]. In the context of repeat surgical treatment for stroke patients, many questions remain unanswered, particularly regarding the incidence and potential mechanisms of perioperative cognitive dysfunction under different surgical conditions [5,22]. The FCT has been widely used to assess symptoms of cognitive dysfunction in rodent models. In this study, we confirmed cognitive impairment in mice receiving TFIF surgery even when cognitive function was not impaired under conditions of prolonged stroke. We found that stroke model mice that underwent TFIF surgery presented abnormal cognitive function at 3 and 7 days after surgery. Neuroinflammation is considered a major cause of cognitive decline in patients with perioperative neurocognitive disorders (PNDs) [23]. Clinical studies have shown elevated levels of peripheral inflammatory cytokines, increased risk of long-term cognitive impairment, and decreased brain function in PND patients [24]. We found significantly increased activation of microglia and increased levels of the inflammatory cytokine IL-1β in the hippocampus of mice with stroke but not in those of mice subjected to TFIF alone. Interestingly, in mice with stroke, TFIF led to increased activation of microglia in the hippocampus and increased levels of inflammatory cytokines, and LPS was detected in the hippocampal tissues of all the mice with a history of stroke who underwent TFIF. These findings indicate that TFIF promotes brain inflammation in mice with stroke.

Alpha diversity and beta diversity are effective and practical indicators of the overall composition of the gut microbiota [25]. We found that stroke and TFIF, whether implemented separately or in combination, altered the beta diversity of the mouse gut microbiota rather than the alpha diversity. In particular, TFIF surgery changed the composition of the gut microbiota at different levels in mice before and after stroke. We identified changes in representative species of the gut microbiota at the genus level. Additionally, compared with those in the control group, nine specific gut bacteria exhibited significant changes in the stroke group. This finding is consistent with previous studies that reported an increase in the abundance of Bacteroides in mice following ischemic stroke [26].

16S rRNA sequencing provides direct evidence of the roles of specific bacteria in diseases and treatment processes [27]. In this study, using multiple strategies, we identified species that were altered due to stroke and/or TFIF, particularly dominant taxa at the genus level. Lactobacillus was decreased in mice with stroke receiving TFIF. In mice without stroke, the abundance of Muribaculaceae_unclassified in the feces of mice receiving TFIF was significantly reduced. Stroke, on the other hand, significantly induced a decrease in Ligilactobacillus in the mice. Given that we used 16S rRNA sequencing, which does not reach the species level, further definition of some bacteria at the genus level is needed.

The composition of the gut microbiota is complex, as we observed changes in dozens of bacteria under different surgical conditions. To elucidate how changes in the gut microbiota under different surgical conditions affect cognitive function, we performed functional predictions with respect to the altered bacterial species and identified a large number of bacteria associated with permeases. Intestinal permeases are multihelix transmembrane proteins that can bind to specific solutes and, through conformational changes, transport the bound solutes to the other side of the membrane. We speculate that the altered gut microbiota composition in mice with stroke following TFIF surgery may impair intestinal defense function and promote intestinal inflammation. Intestinal-derived inflammatory factors such as LPS are often major peripheral factors that contribute to systemic and brain inflammation [28]. We detected a decrease in α-defensin levels in the colon of mice receiving TFIF only after stroke, and these mice presented significantly increased levels of IL-1β and LPS in the colonic villous homogenate. This may be the cause of brain inflammation and cognitive impairment following TFIF in mice with a history of stroke.

While our study provides important insights into the effects of TFIF surgery on cognitive impairment and gut microbiota alterations in mice with a history of stroke, several areas could be further refined in future research. The use of the FCT allowed sensitive assessment of hippocampus-dependent associative learning and memory. However, incorporating additional behavioral tests would enable a broader evaluation of cognitive function, including spatial memory. Additionally, although animal models serve as valuable tools for understanding biological mechanisms, differences in physiology and microbiota composition between mice and humans highlight the need for clinical studies to confirm and extend our findings. Finally, stroke is most commonly observed in older individuals with comorbidities such as obesity, and animal models should ideally reflect these clinical features. However, stroke is increasingly reported in younger populations [29–31]. By using young mice in this study, we aimed to focus specifically on the effects of surgery on cognitive function in stroke survivors while minimizing confounding factors such as aging and obesity that affect cognition.

In conclusion, our study revealed the promoting effect of TFIF surgery on perioperative cognitive impairment in mice with a history of stroke and demonstrated its correlation with alterations in the gut microbiota. These findings shed light on the complex interplay between surgical trauma, the gut microbiota, and cognitive function. By focusing on the gut‒brain axis, this research provides a foundation for future investigations and potential therapeutic strategies to mitigate perioperative cognitive dysfunction in vulnerable populations.

## References

[1] Kong H, Xu LM, Wang DX. Perioperative neurocognitive disorders: a narrative review focusing on diagnosis, prevention, and treatment. CNS Neurosci Ther. 2022;28:1147–67. 10.1111/cns.13873 PMC9253756 35652170

[2] Xu F, Han L, Wang Y, Deng D, Ding Y, Zhao S, et al. Prolonged anesthesia induces neuroinflammation and complement-mediated microglial synaptic elimination involved in neurocognitive dysfunction and anxiety-like behaviors. BMC Med. 2023;21:7. 10.1186/s12916-022-02705-6 PMC9814183 36600274

[3] Cheng C, Wan H, Cong P, Huang X, Wu T, He M, et al. Targeting neuroinflammation as a preventive and therapeutic approach for perioperative neurocognitive disorders. J Neuroinflammation. 2022;19:297. 10.1186/s12974-022-02656-y PMC9743533 36503642

[4] Suo Z, Yang J, Zhou B, Qu Y, Xu W, Li M, et al. Whole-transcriptome sequencing identifies neuroinflammation, metabolism and blood–brain barrier related processes in the hippocampus of aged mice during perioperative period. CNS Neurosci Ther. 2022;28:1576–95. 10.1111/cns.13901 PMC9437242 35899365

[5] Mijajlovic MD, Aleksic V, Stojanovski N, Bornstein NM. Relationship between bone disorders and stroke. Neurol Sci. 2020;41:3579–87. 10.1007/s10072-020-04748-0 33006058

[6] Jacob MA, Ekker MS, Allach Y, Cai M, Aarnio K, Arauz A, et al. Global differences in risk factors, etiology, and outcome of ischemic stroke in young adults – a worldwide meta-analysis: the GOAL initiative. Neurology. 2022;98:e573–88. 10.1212/WNL.0000000000013195 PMC8829964 34906974

[7] Boot E, Ekker MS, Putaala J, Kittner S, De Leeuw FE, Tuladhar AM. Ischaemic stroke in young adults: a global perspective. J Neurol Neurosurg Psychiatry. 2020;91:411–7. 10.1136/jnnp-2019-322424 32015089

[8] Osadchiy V, Martin CR, Mayer EA. The gut–brain axis and the microbiome: mechanisms and clinical implications. Clin Gastroenterol Hepatol. 2019;17:322–32. 10.1016/j.cgh.2018.10.002 PMC6999848 30292888

[9] Gershon MD, Margolis KG. The gut, its microbiome, and the brain: connections and communications. J Clin Invest. 2021;131:e143768. 10.1172/JCI143768 PMC8439601 34523615

[10] Mayer EA, Nance K, Chen S. The gut–brain axis. Annu Rev Med. 2022;73:439–53. 10.1146/annurev-med-042320-014032 34669431

[11] Pan C, Zhang H, Zhang L, Chen L, Xu L, Xu N, et al. Surgery-induced gut microbial dysbiosis promotes cognitive impairment via regulation of intestinal function and the metabolite palmitic amide. Microbiome. 2023;11:248. 10.1186/s40168-023-01689-6 PMC10631187 37936242

[12] Bostick JW, Schonhoff AM, Mazmanian SK. Gut microbiome-mediated regulation of neuroinflammation. Curr Opin Immunol. 2022;76:102177. 10.1016/j.coi.2022.102177 PMC9167715 35462279

[13] Chen C, Liao J, Xia Y, Liu X, Jones R, Haran J, et al. Gut microbiota regulate Alzheimer’s disease pathologies and cognitive disorders via PUFA-associated neuroinflammation. Gut. 2022;71:2233–52. 10.1136/gutjnl-2021-326269 PMC10720732 35017199

[14] Chandra S, Sisodia SS, Vassar RJ. The gut microbiome in Alzheimer’s disease: what we know and what remains to be explored. Mol Neurodegener. 2023;18:9. 10.1186/s13024-023-00595-7 PMC9889249 36721148

[15] Xu X, Hu Y, Yan E, Zhan G, Liu C, Yang C. Perioperative neurocognitive dysfunction: thinking from the gut? Aging (Albany NY). 2020;12:15797–817. 10.18632/aging.103738 PMC7467368 32805716

[16] Liu L, Shang L, Jin D, Wu X, Long B. General anesthesia bullies the gut: a toxic relationship with dysbiosis and cognitive dysfunction. Psychopharmacology (Berlin). 2022;239:709–28. 10.1007/s00213-022-06096-7 35187594

[17] Sun Y, Wang K, Zhao W. Gut microbiota in perioperative neurocognitive disorders: current evidence and future directions. Front Immunol. 2023;14:1178691. 10.3389/fimmu.2023.1178691 PMC10192759 37215136

[18] Pan CL, Dai GL, Zhang HW, Zhang CY, Meng QH, Xu L, et al. Salidroside ameliorates orthopedic surgery-induced cognitive dysfunction by activating adenosine 5’-monophosphate-activated protein kinase signaling in mice. Eur J Pharmacol. 2022;929:175148. 10.1016/j.ejphar.2022.175148 35834964

[19] Qian C, Yang C, Lu M, Bao J, Shen H, Deng B, et al. Activating AhR alleviates cognitive deficits of Alzheimer’s disease model mice by upregulating endogenous Abeta catabolic enzyme Neprilysin. Theranostics. 2021;11:8797–812. 10.7150/thno.61601 PMC8419060 34522212

[20] Myint PK, Poole KE, Warburton EA. Hip fractures after stroke and their prevention. QJM. 2007;100:539–45. 10.1093/qjmed/hcm067 17693418

[21] Poole KE, Reeve J, Warburton EA. Falls, fractures, and osteoporosis after stroke: time to think about protection? Stroke. 2002;33:1432–6. 10.1161/01.str.0000014510.48897.7d 11988628

[22] Evered LA, Silbert BS. Postoperative cognitive dysfunction and noncardiac surgery. Anesth Analg. 2018;127:496–505. 10.1213/ANE.0000000000003514 29889707

[23] Subramaniyan S, Terrando N. Neuroinflammation and perioperative neurocognitive disorders. Anesth Analg. 2019;128:781–8. 10.1213/ANE.0000000000004053 PMC6437083 30883423

[24] Alam A, Hana Z, Jin Z, Suen KC, Ma D. Surgery, neuroinflammation and cognitive impairment. EBioMedicine. 2018;37:547–56. 10.1016/j.ebiom.2018.10.021 PMC6284418 30348620

[25] Guida F, Turco F, Iannotta M, De Gregorio D, Palumbo I, Sarnelli G, et al. Antibiotic-induced microbiota perturbation causes gut endocannabinoidome changes, hippocampal neuroglial reorganization and depression in mice. Brain Behav Immun. 2018;67:230–45. 10.1016/j.bbi.2017.09.001 28890155

[26] Yin J, Liao SX, He Y, Wang S, Xia GH, Liu FT, et al. Dysbiosis of gut microbiota with reduced trimethylamine-N-oxide level in patients with large-artery atherosclerotic stroke or transient ischemic attack. J Am Heart Assoc. 2015;4:e002699. 10.1161/JAHA.115.002699 PMC4845212 26597155

[27] Meng Q, Ma M, Zhang W, Bi Y, Cheng P, Yu X, et al. The gut microbiota during the progression of atherosclerosis in the perimenopausal period shows specific compositional changes and significant correlations with circulating lipid metabolites. Gut Microbes. 2021;13:1–27. 10.1080/19490976.2021.1880220 PMC7954427 33691599

[28] Ghosh SS, Wang J, Yannie PJ, Ghosh S. Intestinal barrier dysfunction, LPS translocation, and disease development. J Endocr Soc. 2020;4:bvz039. 10.1210/jendso/bvz039 PMC7033038 32099951

[29] Hathidara MY, Saini V, Malik AM. Stroke in the young: a global update. Curr Neurol Neurosci Rep. 2019;19:91. 10.1007/s11910-019-1004-1 31768660

[30] Perera KS, de Sa Boasquevisque D, Rao-Melacini P, Taylor A, Cheng A, Hankey GJ, et al. Evaluating rates of recurrent ischemic stroke among young adults with embolic stroke of undetermined source: the young ESUS longitudinal cohort study. JAMA Neurol. 2022;79:450–8. 10.1001/jamaneurol.2022.0048 PMC8922202 35285869

[31] Paul S, Candelario-Jalil E. Emerging neuroprotective strategies for the treatment of ischemic stroke: an overview of clinical and preclinical studies. Exp Neurol. 2021;335:113518. 10.1016/j.expneurol.2020.113518 PMC7869696 33144066

